# Physician Deserts: Navigating the Texas Terrain of Provider Supply and Demand with GIS Mapping

**DOI:** 10.3390/healthcare12232397

**Published:** 2024-11-29

**Authors:** Syed Hussain Jafri, Subi Gandhi, Edward Osei

**Affiliations:** 1 College of Business Administration, Tarleton State University, Stephenville, TX 76402, USA; jafri@tarleton.edu; 2 Department of Medical Lab Sciences, Public Health, and Nutrition Sciences, School of Health and Clinical Professions, College of Health Sciences, Tarleton State University, Stephenville, TX 76402, USA; 3 College of Agriculture and Natural Resources, Tarleton State University, Stephenville, TX 76402, USA

**Keywords:** healthcare access, rural health, health disparities, specialist supply and demand, specialist deserts, physician shortage, GIS mapping

## Abstract

**Background:** Rural health disparities in Texas impact population health due to limited healthcare access, insurance, and transportation challenges, especially in medically underserved areas. A shortage of specialists in rural regions worsens these issues, leading to increased morbidity and mortality rates. **Objective:** Our research aimed to address a knowledge gap by investigating the availability of three medical specialists—cardiologists, pulmonologists, and endocrinologists—in rural counties of Texas and identifying areas where access to healthcare is limited. **Methods:** Utilizing data from regional, state, and federal sources, the analysis geocoded specialist locations and created GIS maps to visualize the distribution of specialists across Texas’s 254 counties. Physician demand was calculated by considering disease incidence and population size, resulting in a county-level physician availability index to highlight areas with shortages. **Results:** Our findings demonstrate a significant deficiency of cardiologists in 196 counties when considering a maximum reasonable travel distance of 50 miles. Comparable deficiencies were observed for pulmonologists and endocrinologists, with western rural counties predominantly comprising the deficiency areas for each specialty. These results emphasize a significant rural–urban disparity concerning access to the three investigated health specialists. **Conclusions:** Addressing geographic disparities can reduce health inequities, improve rural healthcare access, and promote a more equitable healthcare system across Texas. Solutions may include incentives for specialists to work in underserved areas, expanded telemedicine services, and transportation assistance.

## 1. Background

The persistent issue of physician shortages in the United States presents a significant challenge to healthcare delivery, particularly in rural areas where access is already limited. Physician shortages arise when the demand for healthcare providers exceeds the available supply, a disparity that has been worsened by both supply- and demand-side factors [1,2]. On the demand side, factors such as the expansion of Medicaid and Medicare, an aging population requiring increased medical care, and policy changes following the Affordable Care Act have collectively heightened the need for healthcare providers [1,2]. On the supply side, constraints include physician retirements, limited residency positions, and restrictions in graduate medical education programs, which further widen the healthcare access gap [1,2]. Despite national efforts to address physician shortages, rural and underserved regions continue to face this issue more acutely than urban centers, evidenced by disparities in healthcare availability and quality [1,2]. Studies have projected that by 2030, the Southern and Western regions of the U.S. will experience the most severe physician shortages, with deficits expected to reach 92,172 and 63,589 healthcare positions, respectively [1,2]. In contrast, the Northeastern region may see a surplus [1,2]. This regional disparity is particularly evident in Texas, which ranks among the top states facing critical physician shortages. These shortages, especially in rural Texas, create significant public health challenges as many communities face socioeconomic constraints and geographic isolation, further exacerbating healthcare inequities [1,2]. The concentration of specialists in urban centers, coupled with healthcare facility closures in rural areas, leaves residents with limited or delayed access to essential medical care [1,2]. Consequently, rural populations, especially the elderly, experience higher morbidity and mortality rates compared to their urban counterparts [1,2].

In Texas, this issue is particularly pressing. The demand for full-time physician equivalents is projected to increase from 6218 in 2018 to 10,330 by 2032 [1,2]. Although efforts have been made to expand medical schools and residency programs, retaining graduates within the state has proven challenging [1,2]. For instance, between 2000 and 2019, only 49.4% of Texas medical school graduates pursued residency in Texas, and of those, just 59% remained in the state after completing their training [1,2]. These retention issues highlight gaps in current healthcare policies, especially in addressing the needs of rural areas [1,2]. Existing policies often overlook the unique challenges faced by rural communities, failing to adequately address the distribution of specialists [1,2]. Adding to this complexity, many rural hospitals are forced to close due to low patient volumes, high rates of uninsured patients, and inadequate reimbursement policies [1,2]. Texas leads the nation in rural hospital closures, losing 24 hospitals since 2005, which resulted in the loss of 880 hospital beds [1,2]. By 2021, 71 counties in Texas had no hospital facilities, and 11 had no emergency medical services (EMS) stations, forcing residents to travel long distances for basic and emergency care [1,2]. These closures, combined with specialist shortages, worsen healthcare inequities in rural areas, leaving populations vulnerable due to delayed or inaccessible medical services [1,2].

Extensive research has focused on disparities in access to primary care in rural areas; however, the impact of shortages in specialists such as cardiologists, pulmonologists, and endocrinologists on rural health outcomes remains underexplored [1,2]. Specialists play a crucial role in managing chronic conditions prevalent in rural communities, yet there is limited understanding of how their scarcity affects health outcomes in these regions [1,2]. Specialists are crucial for managing chronic conditions like cardiovascular disease, respiratory disorders, and diabetes, which are prevalent in rural communities and significantly contribute to morbidity and mortality [1,2]. Despite the critical role of specialists, most research has primarily focused on access to primary care providers, leaving a gap in understanding how specialist shortages affect health outcomes in rural areas. Addressing the gap in the literature on specialist shortages in rural areas is essential for informing healthcare policies that improve access for underserved populations. While studies have analyzed primary care access, the intersection of specialist shortages and facility closures remains underexplored.

### 1.1. Shortages: Cardiologists

Cardiologists are medical professionals who have undergone specialized education and training in the prevention, diagnosis, and treatment of heart-related conditions. As experts in the field, they possess extensive knowledge of the heart muscle and the blood vessels that transport blood [3]. As cardiovascular diseases are rising due to the aging population, the demand for cardiologists will remain high across all US states [4]. In a recent study on geographic mapping that included 47,225 cardiologists, it was discovered that individuals typically need to travel more than 100 km (about 62.14 miles) to access Heart Failure Cardiologists, unless they reside in major urban areas [4]. In our study, we also demonstrated this disparity in the rural areas of Texas, even though our study did not delve into the types of cardiology subspecialties. Longer distances to cardiologists may decrease the likelihood of seeking medical advice when symptoms first appear, leading to lower compliance with follow-up appointments and causing a delay in treatment for serious conditions. It is important to note that being close to medical services and facilities does not always guarantee access. Although proximity can enhance accessibility, certain social and environmental factors influence accessibility, especially in rural areas [5]. The ambulance response times in rural areas are 12–14 min, twice as long as in urban areas. There will continue to be critical shortages of cardiologists in rural, underserved areas. For example, in 2032, only 56.1% of the demand for cardiologists is projected to be met in the Texas panhandle area [6].

### 1.2. Shortages: Pulmonologists

Pulmonology is a field of medicine that focuses on the diagnosis and treatment of conditions affecting the lungs and respiratory system [7]. Although pulmonary diseases are not as deadly as cardiovascular diseases, chronic lower respiratory diseases are the 6th leading cause of death for Americans [8]. Serious cases with pulmonary diseases will typically require patients to be admitted in critical-care units related to pulmonary diseases such as acute or chronic respiratory failure, chronic obstructive pulmonary disease (COPD), Emphysema, Chronic Bronchitis, Pneumonia and Cystic Fibrosis [9]. A national study [10] demonstrated that around 7% of adults living in urban clusters and 5% in rural areas lacked access to a pulmonologist within 50 miles. This is nearly 3.7 million US adults (1.5 million in urban clusters and 2.2 million in rural areas) who are without a nearby pulmonologist reachable within a 1 h drive.

### 1.3. Shortages: Endocrinologists

An endocrinologist is a medical professional with extensive knowledge in diagnosing and managing illnesses that affect the endocrine system, which comprises the glands and organs responsible for secreting hormones [11]. In a nationwide study [12] with a 20-mile distance radius, it was found that the population-to-endocrinologist ratio within 20 miles was 29,887:1 for adults aged 18–64 years and 6194:1 for adults aged ≥65 years. Further examination of the urban/rural disparity revealed that only 55.5% of adults aged 18–64 and 51.5% of those aged ≥65 years living in rural areas had access to at least one endocrinologist within a 20-mile buffer area, compared to 98.6% and 98.7% of their urban counterparts, respectively. In Texas, specific regions, notably the Panhandle, are forecasted to face a more severe shortage of endocrinologists than other regions. According to projections for 2032, the demand in this region will be met at a rate of only 31.8% compared to public health regions 2 and 3 at 70.9% [6].

### 1.4. Research Objectives

This study aims to address this gap by examining the availability of specialists across all Texas counties and identifying regions with critically limited healthcare access. This research has three main objectives:

1. To analyze the spatial distribution of specialists across Texas to identify counties with limited access.
2. To assess the demand for specialists using demographic and health indicators such as disease prevalence and population density.
3. To utilize these insights to develop evidence-based policy recommendations aimed at reducing healthcare disparities in rural Texas.

By focusing on the role of specialists, this study contributes both to the theoretical framework for evaluating healthcare accessibility in rural settings and to the practical discourse on physician shortages. The insights provided can guide policymakers and healthcare administrators in designing targeted interventions to mitigate healthcare disparities. This research not only addresses an identified gap in the literature but also offers a replicable framework for assessing healthcare accessibility that can be applied to other regions facing similar challenges, ultimately promoting equitable healthcare distribution.

## 2. Methods

We calculated the demand and supply for specialists who are associated with treating some of the leading causes of morbidity and mortality in the United States, namely cardiovascular outcomes (cardiologists), diabetes (endocrinologists), and chronic lower respiratory diseases (i.e., pulmonologists). We calculated the county-level demand and supply of these specialist services for patients residing in Texas.

To meet the goal of this objective, we took the following steps:

1. Estimate the number of specialists available in each county using data from the Texas Medical Board.
2. Estimate the demand for specialists in each county by analyzing the MONAHRQ data of the Texas Department of State Health Services (DSHS).
3. Use Geographic Information System (GIS) maps to identify areas where there are surpluses or shortages of selected specialists in Texas.
4. Propose policy recommendations to address the gap between the needs and availability of specialists, particularly in rural counties of Texas.

### 2.1. Data Sources

A variety of data sources were utilized to obtain the necessary variables for calculating physician supply and demand for different counties, as explained below:

(a) Data provided by Texas Health Erath County were utilized to predict the number of patient visits by specialty in the Stephenville region based on projections of physician requirements derived from a contract with Nielsen, Inc., New York, NY, USA.
(b) Aetna Inc. database was used to garner physical addresses of physicians by specialty.
(c) The Texas Medical Board’s physician licensure file from March 2016 was used to determine the supply of physicians by location. These data were employed to estimate the number of full-time equivalent physicians practicing within each county.
(d) The Texas Department of Health and Human Services data for 2008 to 2014 were used to obtain information on inpatient hospital discharges by disease code.
(e) Texas (MONAHRQ) 2012 hospital data were used to assess utilization and quality.
(f) The USDA Economic Research Service rural–urban continuum codes were used to identify counties as rural or urban.
(g) Population census figures at the county level, along with the MONAHRQ data and patient visit information, were used to estimate the demand for physician services.
(h) Texas Behavioral Risk Factor Surveillance System, Texas Center for Health Statistics, and other publicly available national databases. This dataset provides additional insights into disease incidence.

To address missing data and potential biases, this study incorporated several strategies to ensure data completeness and reliability. When specific variables lacked data, imputation methods were employed, such as averaging values from nearby counties with similar demographic profiles, to fill gaps. Potential biases were considered, particularly those related to the use of proxies like disease incidence for estimating physician demand. For example, relying on disease incidence data assumes that all individuals diagnosed with a condition will seek specialty care, which may not accurately reflect actual healthcare-seeking behavior. This approach aims to provide greater transparency regarding the limitations and assumptions inherent in the methodology. The selection of data sources was guided by their relevance to the study objectives. The Texas Medical Board provided precise, county-level data on physician availability, crucial for understanding the distribution of healthcare providers. In addition, MONAHRQ data were utilized for its detailed county-level statistics on disease incidence, allowing for an estimation of the need for specialty care based on localized health trends. These sources were chosen to ensure a robust and accurate assessment of both the supply and demand for healthcare providers across Texas, aligning with the study’s focus on identifying gaps in specialist availability.

### 2.2. Rural/Urban County Designations

Counties were designated as rural or urban based on the 2004 USDA-ERS rural–urban county typology codes [13]. According to our analysis, 85 of the 254 counties in Texas were designated as rural, based on a rural–urban continuum value of 7 or greater. Given this rural/urban classification, the following methodology was used to compute and contrast a county-level index of health provider availability relative to needs for rural and urban areas for each of three medical specialties: cardiovascular, pulmonary, and endocrinology.

### 2.3. GIS and Gravity Modeling

This study employs Geographic Information System (GIS) models to provide a detailed analysis of the supply and demand for healthcare providers, considering demographic and socio-economic variables such as race, class, ethnicity, income differences, rural and urban designations, and travel cost variations due to terrain, traffic, and transportation expenses. The methodology integrates GIS mapping with supply and demand indices to analyze specialist availability, an approach proven effective in previous healthcare access research. Prior studies have utilized GIS mapping to address physician shortages by spatially analyzing healthcare access, particularly for primary care physicians in underserved areas [14]. However, the application of this method to examine shortages of specialists—such as cardiologists, pulmonologists, and endocrinologists—remains relatively sparse in the existing literature [15]. Traditional models have often relied solely on physician counts within geopolitical boundaries, which may neglect the healthcare needs of populations just outside these areas. To overcome these limitations, this study implements a modified gravity and floating catchment area model, incorporating distance decay functions and adjustments for population density and economic activity. This approach enhances the estimation of accessibility by accounting for factors such as travel distance and population density, thus offering a more refined analysis of healthcare accessibility beyond simple location counts. The gravity model, often used in healthcare access studies, calculates physician availability based on proximity and population density [16]. Specifically, the model assigns greater weight to physicians who are closer to a county’s population center and to those practicing in areas with higher population densities. For example, a cardiologist practicing within 10 miles of a rural county will contribute more to that county’s supply score than one located 50 miles away [17,18]. This approach allows us to estimate how easily residents can access specialists in their area, reflecting both distance and demand. These clarifications are designed to make the methods section more accessible to non-specialist readers while maintaining the rigor of the analysis.

GIS mapping is a powerful tool that provides spatial insights into the distribution of healthcare services. While it has been extensively applied in urban healthcare access studies, its use for assessing specialist shortages in rural areas has been limited. By leveraging GIS-based spatial analysis, this research evaluates healthcare access across Texas counties, focusing specifically on specialist availability. The use of gravity models enables a nuanced assessment of regions with critical shortages by considering both geographic and socio-economic factors, ultimately helping to identify areas where healthcare resources are insufficient relative to population needs. As such, a GIS database that catalogs medical providers by location was developed for the entire state based on the geocoded physical address of each medical specialist. To determine the supply of physicians in each county, we used a GIS-based gravity model [19], which we motivate presently.

Most published physician supply estimates were derived as simple counts of physicians based on practice location—a procedure we refer to as simple location counts. With simple location counts, physician supply to an area is estimated as the number of physicians located within the geopolitical boundary of that area, regardless of proximity to the boundary. Thus, if there are, for example, n Cardiologists located in an area of interest would constitute the Cardiologist’s physician supply estimate. This number is derived by geocoding physician practice addresses widely available in physician licensure files to determine how many are located within relevant geopolitical boundaries. Unfortunately, simple location counts disregard many spatial dimensions of health care delivery since these counts are based solely on whether the physician is within the boundary of the area of interest. Physicians serve patients who live outside the geopolitical boundary of the county, state, or service area where their practice is located.

A more appropriate definition of physician supply is the number of FTEs of physicians of the given specialty that are available to provide direct patient care around the area of interest. The location of physicians relative to arbitrary geo-political boundaries is no longer of interest, but as we shall see, a more pertinent consideration is proximity to residents, that is, proximity to the population centroid of the area of interest. For simplicity, we shall assume here that the area of interest is a county. The analysis presented here is readily transferable to other definitions of the area of interest—province, state, congressional district, zip code, and so on.

By using FTEs instead of counts, we allow for differences in work effort among physicians. Male physicians tend to work more hours than female physicians [6], and physicians of different cohorts also contribute various levels of effort to direct patient care. For the following discussion, we will simplify the exposition by dropping the reference to time. All variables indicated below refer to the same time, a typical calendar year.

Total location FTEs of physicians located within each county are an improvement over simple location count. They are computed by adjusting the location counts using data on hours of work effort. The distribution of physician weekly hours of work by gender indicates a definite difference in work effort preferences between male and female physicians, necessitating adjustment of location counts by work effort to estimate physician supply more accurately. Total physician FTEs are obtained by dividing the number of hours worked per week by each of the physicians by the average number of hours worked per week by all physicians and summing up across all the $L_{j}$ physicians, where $L_{j}$ represents the number of physicians of the chosen specialty in county j. Consequently, the total FTEs of physicians located in county j, $f_{j}$ is computed as:

(1) $$f_{j}=\sum_{i=1}^{L_{j}}h_{i}/\underline{h}$$

where $h_{i}$ is the hours worked in a typical week by ith doctor of this specialty located within the county as reported in the database and $\underline{h}$ is the average number of hours worked per week by all physicians of the chosen specialty.

As stated above, physician location FTEs, represented by $f_{j}$, are an improvement over simple location counts because they allow for various levels of work effort among physicians. However, they still do not account for accessibility outside the geopolitical boundary of the physician’s place of practice. Further adjustments to capture healthcare delivery outside arbitrary political boundaries are possible through floating catchment or gravity models. Two-step (2SFCA) [20,21,22,23] and three-step (3SFCA) [18,24] floating catchment area models were proposed as augmentations that adjust physician supply estimates based on the distance of patient travel to the physician’s practice location. With standard 2SFCA and 3SFCA models, physicians are assumed to supply their services equally to all patients within a predetermined maximum feasible travel distance or catchment radius. Thus, these models address a major limitation of simple location counts and location FTEs by allowing physician supply to extend beyond geopolitical boundaries.

Notwithstanding their superiority to simple location counts and location FTEs, standard 2SFCA and 3SFCA models of physician supply estimation are limited in that they do not allow for a reduction in physician supply as the distance of travel increases within the catchment region. Recent augmentations of these models do address this limitation with distance decay functions. However, there is no consideration of physicians supplying their services to catchments or areas with higher population density or economic activity. In this study, we employ a modification of the gravity and floating catchment models used in other studies [19] to address these limitations. Specifically, our approach incorporates distance decay functions and allows supply to increase with increasing population density or greater economic activity.

To introduce our model, we start with the location counts given above and proceed by using a three-step process. First, we calculate each physician’s FTE based on hours worked as compared to the average hours of physicians of that specialty. In this step,

(2) $$f_{i}=\frac{h_{i}}{\underline{h}}$$

where $f_{i}$ is the total FTE of physician i, $h_{i}$ are hours worked by this physician and $\underline{h}$ is the typical number of hours worked by the average physician of this specialty.

In the second step, we compute the proportion of each physician’s FTEs that are available to patients in each county. In this step, we include only physicians located within a predetermined maximum feasible travel distance (or catchment radius) for patients within each county. This step is the crux of the gravity model. The proportion of the FTE of physicians i that is made available to residents in each county j is expressed in direct proportion to the total population (or a measure of economic activity) of county j (if its population centroid is within the maximum travel distance) and inversely proportional to the distance between the physician’s practice location and the population centroid of county j. With a continuous linear distance decay parameter, we have

(3) $$w_{ij}=\frac{p_{j}/d_{ij}}{\sum_{j=1}^{n}\left[\frac{p_{j}}{d_{ij}}\right]}\;\;\;\;\;\mathrm{if}\;{d_{ij}\leq d}_{MAX}$$

$$w_{ij}=0\;\;\;\;\;\;\mathrm{otherwise}$$

where $w_{ij}$ is the proportion of physician i’s FTE that is available to county j (we use $w_{ij}$ below as weights in estimating physician availability), $d_{ij}$ is the distance from practice location of physician i to population centroid of county j, $p_{j}$ is the population of county j, and $d_{MAX}$ is the maximum feasible travel distance of patients to a physician of this specialty (essentially the radius of the catchment area). By definition, $\sum_{j=1}^{n}w_{ij}=1$. In this study we assume $d_{MAX}=50$ miles based on input from Texas Department of State Health Services officials who are familiar with patient travel distances for care. In this study, a 50-mile radius was selected as the threshold for acceptable travel distance for patients to access specialists. This decision is rooted in accessibility considerations common in rural health literature [10,19,20], where distance is a critical factor in healthcare access. Previous studies have noted that residents in underserved areas often perceive 50 miles as the upper limit for feasible travel to receive medical care [17,18]. Additionally, this threshold aligns with benchmarks used in other studies on physician accessibility in rural settings, which similarly recognize the 50-mile radius as a realistic boundary to assess healthcare availability in areas with sparse specialist distribution.

Higher weights are assigned to locations closer to the physician’s practice address or with higher population densities. In the special case where the population distribution within the feasible travel distance is even, the weights reduce to

(4) $$w_{ij}=\frac{1/d_{ij}}{\sum_{j=1}^{n}\left[\frac{1}{d_{ij}}\right]}$$

In that case the distribution of a physician’s FTE would be cone-shaped with maximum weight at the practice location (the apex of the cone), and total cone volume equal to $f_{i}$. For a physician working full-time, the total volume of the cone would be $f_{i}=1$. The weights could alternatively be calculated using other measures, such as total income instead of population. Furthermore, Equation (4) can be used to compute the weights if it is not desired to adjust for population or an income or economic activity variable. In the application below, we use (3) to compute the measure of physician supply (with population adjustments).

In the last step, we sum up the resulting FTE proportions across all physicians, each physician’s FTE being weighted by the corresponding $w_{ij}$ to obtain a robust estimate of physician supply to residents of county j:

(5) $$s_{j}=\sum_{i=1}^{L_{j}}f_{i}w_{ij}$$

where $s_{j}=$ supply of physicians to residents in county j, and n= total number of physicians.

Our preferred measure of physician supply is $s_{j}$, using the weights defined in (3). In the availability indices presented below, we use $s_{j}\;$in conjunction with a physician demand estimate.

### 2.4. Calculation of Physician Demand

We defined physician demand as the total FTEs of specialists required based on disease incidence and the county’s population size. By far, the best data available on physician demand are the frequency of visits to specialists. Where available, data on the frequency of patient visits to physicians of a given specialty constitutes physician demand for that specialty. Where data are not available, physician demand can be estimated by proxy based on disease incidence and frequency of visits data from similar locations.

The Texas MONAHRQ database provides disease incidence at the county level. For each county and specialty, disease incidence expressed as the number of patients with a given disease condition per thousand of the population was used as a proxy to determine the relative need for services for the corresponding physician. This disease incidence figure was used to estimate the frequency of patient visits based on data in a reference county for which patient visit data exists.

Given the MONAHRQ data, we estimated physician demand as follows. For each county j, the frequency of patient visits in a reference county r for which we have physician demand data, was used to determine demand for physicians of that specialty in any given county j as:

$$d_{j}=\frac{e_{j}}{e_{r}}v_{r}$$

where

$d_{j}$ is the demand for specialist services in county j,

$e_{j}$ is the disease incidence in county j,

$e_{r}$ is the disease incidence in the reference county r, and

$v_{r}$ is the frequency of patient visits to physicians of this specialty in the reference county r.

Using this method, the demand for physician services was constructed for each county and for each of the three specialties included in the study.

Given the foregoing, each county’s demand and supply estimates were used to construct county-level physician availability indices. These indices were computed simply as the ratio of supply to demand:

(6) $$a_{j}=\frac{s_{j}}{d_{j}}$$

where $a_{j}$, $s_{j}$, and $d_{j}$ are, respectively, the county-level availability index, supply, and demand of physicians of the given specialty in county j.

Based on our construction, availability index values greater than 1 indicate a surplus or an overabundance of healthcare providers, while index values less than 1 indicate a deficit or shortage of medical providers.

## 3. Results

### 3.1. The Demographic and Risk Drivers of Specialists Supply in Texas

**Figure 1:**
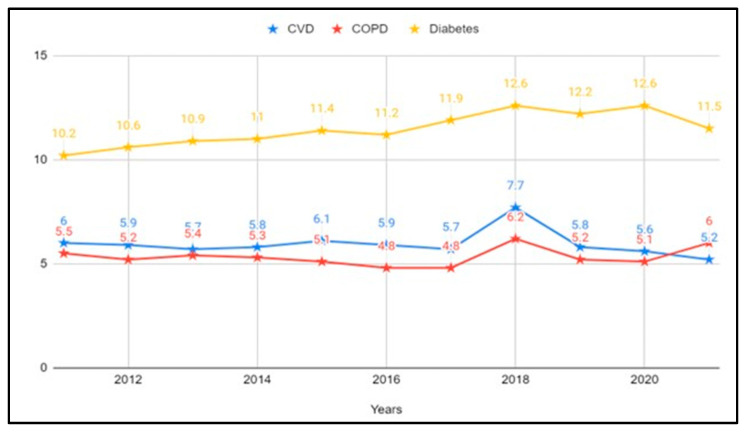
Prevalence of CVD, COPD, and diabetes (2011–2021). Data source: Texas Behavioral Risk Factor Surveillance System, Texas Department of State Health Services [25].

**Figure 2:**
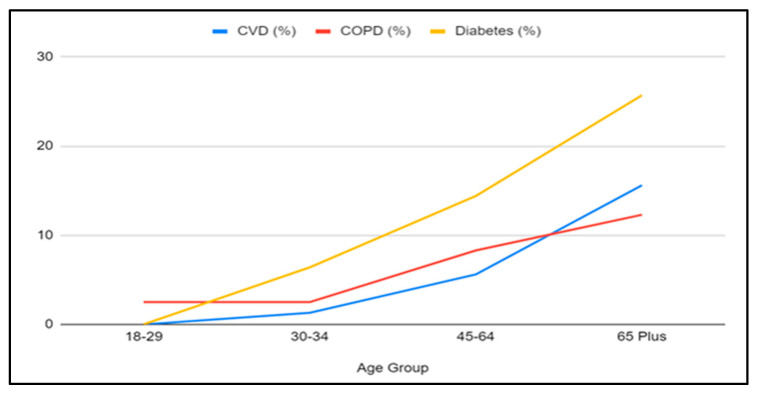
Prevalence of CVD, COPD, and diabetes among various age groups in TX (2021). Data source: Texas Behavioral Risk Factor Surveillance System, Texas Department of State Health Services [25].

To provide a preliminary insight into disease incidence and demographic drivers, we used the Texas Behavioral Risk Factor Surveillance System Data [25] to evaluate the prevalence of cardiovascular disease (CVD), Chronic Obstructive Pulmonary Disease (COPD), and Diabetes from 2011 to 2020 in the state. Additionally, we investigated specific age groups that exhibit a disproportionate burden of these conditions compared to others. The number of individuals affected by cardiovascular, pulmonary (specifically COPD), and diabetes remained relatively stable between 2011 and 2021 (see Figure 1). Not surprisingly, the age group most at risk for all three conditions is individuals aged 65 years and older (see Figure 2).

**Figure 3:**
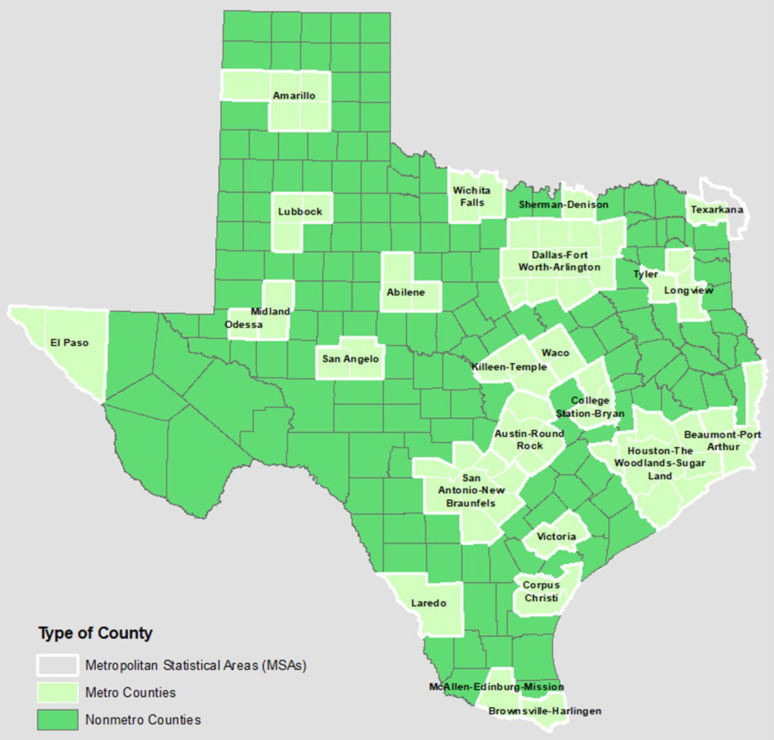
Texas metro and non-metro counties [26].

**Figure 4:**
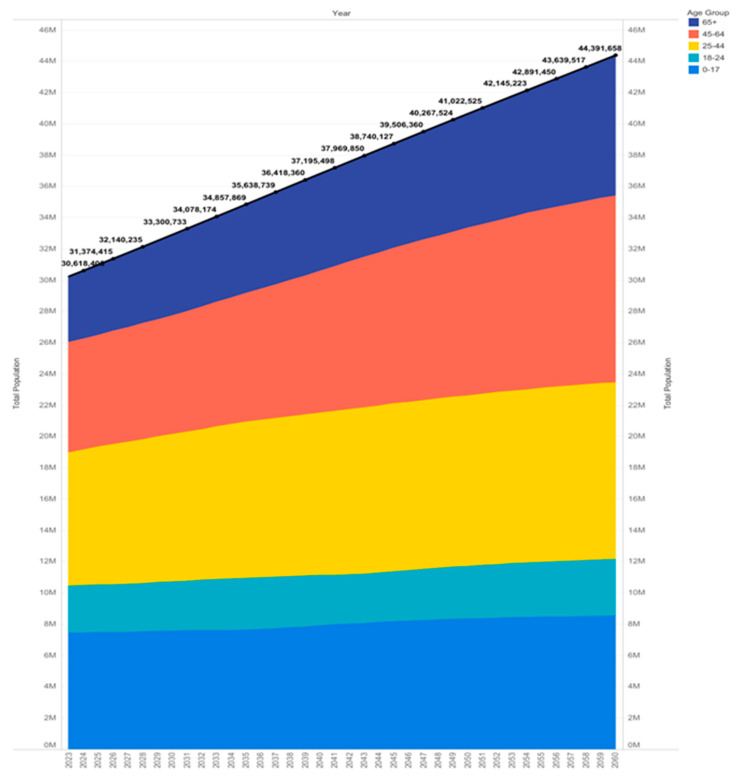
Population growth projection for Texas (2023–2060) [28].

Rural counties make up over 60% of Texas (Figure 3), with approximately 19% of the state’s population residing in these areas [26,27]. Recently, the population threshold for qualifying as an urban area was raised from 2500 to 5000, reclassifying 114 of Texas communities as rural. Between 2023 and 2050, the demographic cohorts experiencing the most rapid growth will be individuals aged 65 and above. During this timeframe, the population of individuals aged 65 and older is projected to expand by over 88%, as displayed in Figure 4.

### 3.2. Specialty-Specific Disparities

#### 3.2.1. Case I. Cardiology

**Figure 5:**
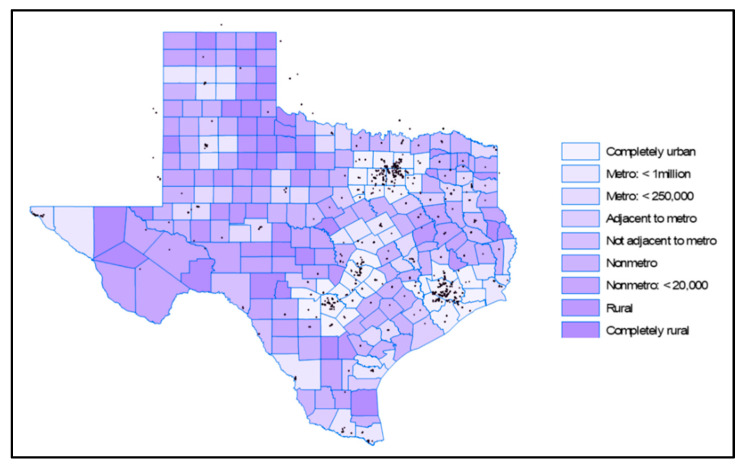
Distribution of cardiologists in and around Texas counties: 2015.

The determination of the provider availability index is contingent upon the distance that a patient can travel to consult with a specialist. In our analysis, out of the 254 counties in Texas, 58 counties showed an overabundance of cardiologists if the benchmark for acceptable travel distance was established at a 50-mile radius, as indicated above. Those counties are primarily clustered around the major cities and in the counties close to Houston, Dallas, and Fort Worth metroplexes and along the Austin–San Antonio corridor. In contrast, the remaining 196 counties, most of them rural in the western part of the state, showed a deficiency in cardiologist availability. If the extent of travel were limitless, specifically if patients could reach a specialist in any part of the state with relative ease, the tally of counties with an ample number of cardiologists would rise to 77, whereas the number of counties experiencing a shortage would reduce to 177. Furthermore, multiple counties in the rural western and southwestern parts of the state have contiguous countries without cardiologists, signifying a critical shortage. The distribution of cardiologists in Texas by location of practice is presented in Figure 5, with each dot representing one cardiologist.

#### 3.2.2. Case II: Pulmonology

**Figure 6:**
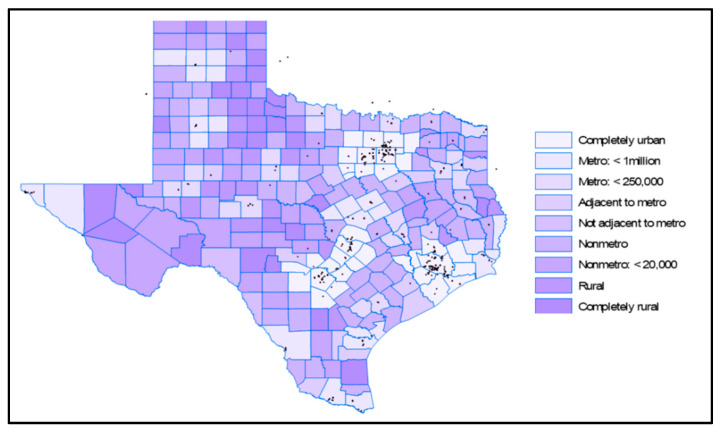
Distribution of pulmonologists in and around Texas counties: 2015.

In contrast to the high concentration of cardiologists depicted in Figure 5, the number of pulmonologists in Texas is significantly lower, as illustrated in Figure 6. Pulmonologists, similar to cardiologists, tend to establish their practices predominantly in large urban centers, resulting in a corresponding scarcity of their presence in rural areas. Our study suggests that Texas residents need 1052 pulmonologists but currently have about 730 in active practice, indicating a specialist shortage. That is, there is a demand for 1052 FTEs of pulmonologists, but only 730 FTEs are available annually. Further, our analysis revealed that six counties lack pulmonologists within a 100-mile radius. In particular, certain rural counties like Presidio County do not have a pulmonologist available within approximately 170 miles from the county’s centroid in Texas. While metropolitan and East Texas counties exhibit slightly better access to pulmonologists compared to West Texas counties, some East Texas counties are in proximity to medical facilities, resulting in high availability indices. The data in Figure 6 represent the distribution of pulmonologists in Texas by location of practice, with each dot representing one pulmonologist. In urban areas, where there are multiple practitioners, some dots may overlay each other.

#### 3.2.3. CASE III: Endocrinology

Figure 3 displays the dispersion of endocrinologists throughout the state, confirming the anticipated pattern of their concentration in the major urban areas of the state, similar to cardiologists and pulmonologists (Figure 5 and Figure 6). Rural counties situated in proximity to these urban areas have good access to cardiologists.

**Figure 7:**
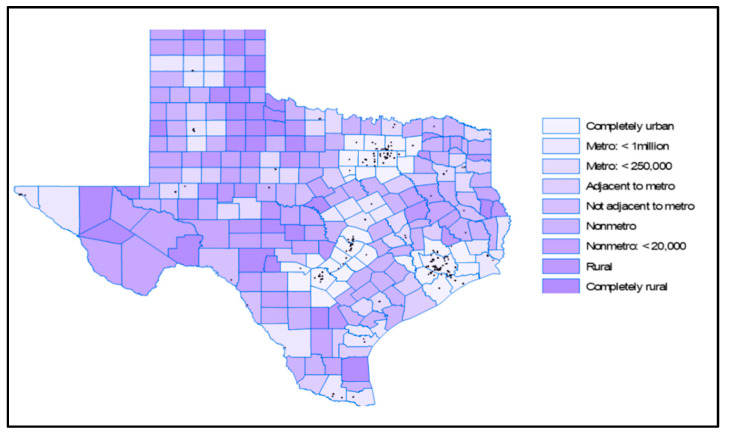
Distribution of endocrinologists in and around Texas counties: 2015.

Our analysis found that the healthcare availability in rural Texas counties varied markedly depending on the county’s location. Health-care provider availability/needs indices range from 0.1 to 4.9, with an average of 0.6 if the feasible travel distance is limited to 50 miles. The analyses suggest that some rural counties are well-represented in terms of medical specialists because of their proximity to specialists in neighboring metropolitan centers (a.k.a. closer to urban areas). However, in several rural counties, especially in the sparsely populated western counties in Texas, residents in areas with limited or no access to specialized care will incur significant costs in traveling and bear other substantial expenses. The distribution of endocrinologists in Texas by location of practice is presented in Figure 7, with each dot once again representing one endocrinologist.

## 4. Discussion

Physician scarcities within the United States pose a significant concern, with far-reaching ramifications for both individual welfare and the nation’s healthcare infrastructure. Similarly, as a state, Texas is falling short in meeting the needs of patients, as they are experiencing extended wait times while doctors are encountering burnout. A significant majority (225 out of 254) of counties in the state are designated as Health Professional Shortage Areas (HPSA), underscoring the challenge in these areas within which a total of 6,066,420 Texans reside [29]. The findings of our investigation revealed a significant shortage of cardiologists, pulmonologists, and endocrinologists overall in Texas, particularly in rural and underserved areas where Emergency Medical Services (EMS) and healthcare access are scarce. On average, the EMS takes 7 min to arrive on the scene in an urban area after receiving a 911 call. In rural areas, this median time extends to over 14 min, and nearly 1 out of 10 incidents have to wait for almost 30 min for EMS personnel to arrive [30]. These issues are particularly worrisome given the crucial role these specialists play in the healthcare of older adults. The presence of limited healthcare services and various social and geographic barriers can exacerbate these impacts, especially due to the increasing population of elderly individuals residing in rural regions of Texas in comparison to urban areas [28,31,32,33]. This situation exposes them to distinct challenges related to aging in rural areas, as these locations are not equipped to adequately address the disparities associated with aging in rural communities [5,34,35]. As Texas experiences rapid population growth [28], it is likely that more uninsured and underserved individuals will fall through the cracks and face premature death if the supply and demand ratios are not narrowed within a decade for these specialties.

A recent report by the Texas Department of Health and Human Services [6] estimated the availability and need for diverse types of physicians in 2018 and predicted the supply and demand for various specialties in 2032. The findings demonstrate a significant shortage of specialists, with only 72% of the demand for cardiologists being met, followed by 72.1% for endocrinologists and 81.4% for pulmonologists. The failures of rural hospitals in general and other hospitals undergoing financial difficulties as well as closures will pose challenges for the severely serious patients, requiring hospitalization and, in addition, have critical care units along with the sub-specialist physicians trained to treat them [22,23].

### 4.1. Potential Alternatives to Bridge the Shortage Gap of Specialists

This study identifies critical shortages in specialist availability across rural Texas, where patients face significant barriers to essential care due to geographical and economic challenges. Based on the study’s findings, several potential strategies can be considered to mitigate these shortages. The GIS-based analysis revealed the impact of geographic proximity on healthcare access, suggesting that expanding telehealth services supported by improved broadband infrastructure could alleviate travel burdens for rural patients, thus enhancing access to specialist care. Additionally, addressing the concentration of specialists in metropolitan areas may require expanding medical education programs and residency slots, along with creating incentives for graduates to serve in rural regions. The results also emphasize the need for a more flexible healthcare workforce, utilizing physician assistants (PAs) and nurse practitioners (NPs) to provide complementary care where specialist access is limited. These alternatives align with the study’s supply–demand indices, which highlight the disparities in healthcare access due to uneven specialist distribution. While these proposed solutions are informed by the spatial analysis conducted, integrating them into practice would require aligning healthcare policies with the identified gaps. By leveraging telehealth and expanding the healthcare workforce, it may be possible to address the spatial and systemic disparities highlighted by the study’s methodology, ultimately promoting a more equitable distribution of care across Texas. Further discussion is needed to explore how these strategies can be effectively implemented in alignment with the study’s empirical findings.

#### 4.1.1. Telehealth

Telehealth and the use of the internet are growing in importance, generally and specifically, to reach rural and underserved populations. Between July and December of 2022, 58.5% of adults sought health or medical information on the internet. This trend was more pronounced in women than in men [36]. The use of the internet, either to access information about health conditions today or to use telehealth, is correlated with one’s overall ability to possess digital literacy and their community’s ability to have broadband coverage. On both these counts, unfortunately, the rural residents and communities are lagging [37]. Also, lack of technical means and language barriers as well as digital literacy issues, can complicate the use of telemedicine for physicians [38]. There is a need for establishing and delivering training programs in rural areas through the network of community public health officials, regional universities and colleges, and extension agents [39].

#### 4.1.2. Broadband Access

Access to high-speed internet increases the volume of information accessible in healthcare markets. Access to high-speed internet is highly correlated with digital access, which is recognized as one of the determinants of health [40]. The availability of online information has the potential to enhance health outcomes by mitigating information asymmetries, enabling patients to select superior-quality healthcare providers, or incentivizing providers to enhance the quality of their services [41]. Unfortunately, rural communities are found to be trailing behind their urban counterparts in terms of broadband access, and Texas is no exception [42,43]. To bridge the gap, recently federal funding has earmarked USD 65 billion (about USD 200 per person in the US), with almost 67% of this amount allocated for the Broadband Equity Access and Development (BEAD) program, which will expand broadband access, especially in rural areas [44,45]. Texas is also expected to gain over USD 3.3 billion (about USD 10 per person in the US) in federal funding to support a significant expansion of broadband infrastructure. This aid is expected to result in a groundbreaking allocation of resources in an area where around 7 million residents are deprived of high-speed internet connectivity. However, uncertainties remain in how much federal dollars can be stretched to cater to most underserved Texans with this alternative for connectivity [46].

#### 4.1.3. Medical Graduates

Admittance to Graduate Schools in Medicine can be tailored to offer preferences to those who commit to serving in underserved areas once they complete their training. For areas that face severe shortages, the State Department and Department of Homeland Security can increase the J-1 visa quota to allow foreign medical graduates to practice in the United States with a commitment to serve in rural areas [47]. Additionally, the state health department can implement the Conrad 30 J-1 visa waiver program that allows 30 International Medical Graduates (IMG) annually for a waiver of the J-1 visa obligation, mandating them to return to their country of origin for a period of two years [48]. Another long-term solution that can be explored to increase enrollment in both medical education and residency programs is to invest in building the necessary infrastructure and faculty resources. According to a report from the Texas Higher Education Coordinating Board [49], in 2019, Texas funding for Graduate Medical Expansion (GME) was a mere USD 2.85 million. Recognizing the need for additional physicians, public funding increased to USD 150 Million in the 2020–2021 biennium year and has exceeded more than USD 200 million for the last four years to expand medical education and first-year residency programs [49] According to this report, GME Expansion Program has supported the creation of 508 new first-year residency positions between 2014 and 2023. Even though the GME expansion will narrow the gap between supply and demand for physicians, the rising population in Texas necessitates additional programs to address these demands. According to recent data, Texas is adding 1000 individuals to the state every single day [50], indicating the scope of the demand for physicians and specialists.

#### 4.1.4. Physician Assistants

The landscape of health and medical care is changing with the increased use of physician assistant/associate (PA)-guided and supported professionals. Ever since Dr. Eugene Stead initiated the first academic program at Duke University in 1965, the presence and utilization of PAs have grown exponentially [51,52]. The concept of a physician assistant was conceived to address the shortages of primary care physicians and has now evolved in the practice of specialists as well, which has narrowed some of the service gaps in healthcare. A recent report from Texas Tech University highlights the importance of the utility of PAs, mainly in the rural areas in Texas [53]. However, the distribution of PAs is low per 100,000 population compared to other states such as Alaska, Pennsylvania, Connecticut, North Carolina, and Montana [54]. Allowing PAs to manage their practice in rural areas can work in bridging gaps between disparities and healthcare. This could incentivize them to make long-term commitments to underserved areas and provide high quality of care to patients [55].

#### 4.1.5. Other Health Professions

Nurse practitioners have also become an integral part of delivering healthcare. In 2023, the United States had over 385,000 licensed nurse practitioners, who have become essential in healthcare delivery [56]). A national study of the role of nurse practitioners concluded that the growing nurse practitioner supply is filling the gap in offsetting low physician supply, especially in underserved and rural areas [57]. A recent study by the Temple Foundation has discovered that Texas has the potential to reduce the shortage of primary care providers by 32% through the elimination of a state requirement on Advanced Practice Registered Nurses (APRNs), such as nurse practitioners. This requirement mandates that APRNs must establish and uphold a contract with a physician to be able to practice. Furthermore, the removal of these limitations could result in the creation of numerous employment prospects and potentially save the state of Texas up to USD 47.7 million in the first two years alone [58,59].

Community Health Workers (CHWs) serve as essential liaisons between healthcare professionals and patients, especially in rural and underserved communities [60]. CHWs enhance healthcare results through the facilitation of healthcare accessibility, the contribution of significance to the healthcare workforce, and the enhancement of the well-being of their patients and service users, particularly individuals residing in underserved areas with marginalized racial and ethnic groups [61]. CHWs are playing a vital role in many preventatives and disease management for patients in Texas [60,62]. Recognizing the need for CHWs in the state, many health agencies and universities have launched CHW training centers and certification programs [63]. However, some CHWs face dissatisfaction with organizational support, job security, or pay, as they are one of the lowest paid members of the healthcare workforce [64].

Community Paramedicine (CP) programs are becoming popular in rural communities as they remain a bridge between primary care and emergency care [65]. Paramedicine is a form of practice and health occupation that specializes in a variety of environments, including urgent situations and primary care [66]. Rural residents have inadequate access to 911 and other emergency services, which could strain the EMS and healthcare systems. Community paramedics can fulfill public health and primary care roles to address the healthcare needs of rural residents [67]. A survey conducted in 2017 unveiled that there are at least 129 CP programs in the United States, with more than 40% of them offering services in rural regions [68]. These programs have been instrumental in helping patients manage chronic disease conditions and in reducing ER visits for patients [68], among other things. There are numerous organizations and institutions in Texas that provide paramedicine programs, both in an online and in-person format [69,70,71,72].

### 4.2. Strengths of Our Study

The study highlights significant disparities in the availability of specialists, particularly cardiologists, pulmonologists, and endocrinologists, between rural and urban counties in Texas. These findings align with prior research indicating that physician shortages are more pronounced in rural areas due to factors such as geographic isolation, lower population densities, and limited healthcare infrastructure [73,74]. However, the use of GIS-based gravity modeling in this study provides a more nuanced understanding of specialist distribution, highlighting regions with critical healthcare gaps that traditional methods might overlook [18].

The existing literature has primarily focused on shortages of primary care providers, with limited exploration into specialist access in rural settings [19]. Our analysis demonstrates that rural Texas counties face a pronounced deficit in specialists, forcing residents to travel long distances to access specialized care. This travel burden can significantly delay diagnosis and treatment, particularly for chronic conditions like cardiovascular disease and diabetes, contributing to higher morbidity and mortality rates among rural populations [75]. Comparatively, urban areas such as Houston and Dallas-Fort Worth exhibit sufficient numbers of specialists, whereas rural areas remain underserved, exacerbating existing healthcare inequities [76].

We also demonstrated that 77% of rural counties lack adequate pulmonologist coverage, which is consistent with national trends indicating specialist shortages are most acute in less densely populated areas [20]. The GIS analysis identified specific counties with the most severe deficits, suggesting that targeted policy interventions, such as expanding telehealth services and incentivizing specialists to practice in underserved regions, could alleviate these disparities [17].

### 4.3. Limitations of Our Study and Future Directions

Several limitations warrant consideration. The use of proxies for estimating physician demand, such as disease incidence rates, may not accurately capture actual healthcare utilization. This approach assumes that all diagnosed individuals seek specialty care, which may not always hold true due to barriers like transportation, lack of insurance, or health literacy [77]. Additionally, GIS gravity models, although effective in mapping supply and demand, may not fully account for socioeconomic factors such as income disparities and digital access, which significantly influence healthcare accessibility [24].

Integrating patient-level data in future studies could provide a more accurate picture of healthcare-seeking behavior, particularly in rural areas. Real-time updates on physician practice locations and socioeconomic variables would further refine accessibility estimates. The findings suggest that expanding telemedicine services and creating financial incentives for specialists to practice in rural Texas are crucial steps toward addressing healthcare inequities [78].

## 5. Conclusions

Physician availability, particularly for specialists, remains a critical issue in rural areas. This study highlights the specific challenges faced in rural Texas, focusing on specialties like Cardiologists, Endocrinologists, and Pulmonologists. Several barriers prevent these specialists from establishing practices in these regions, including demographic shifts toward an aging population, persistent poverty, inadequate transportation infrastructure, limited internet access, low health insurance coverage, and high unemployment rates. These factors collectively create economic disincentives, making it less attractive for specialists to practice in rural areas. This situation exemplifies a classic case of market failure, where the private healthcare market alone is insufficient to meet the needs of underserved communities, thus requiring proactive government intervention. To address these disparities, the study proposes several targeted policy measures. These include reallocating state and federal resources to prioritize rural healthcare and offering economic incentives—such as tax breaks, loan forgiveness, and increased reimbursement rates—to encourage specialists to serve in underserved areas. Expanding telemedicine infrastructure is also recommended to overcome the geographic and transportation barriers that hinder access to specialty care.

These policy recommendations are essential for improving healthcare access and outcomes in rural communities, where the lack of specialized medical care contributes to poorer health outcomes. Ensuring equitable healthcare access in these areas not only supports the well-being of rural populations but also strengthens the overall public health framework. The health and vitality of these communities are critical to the nation’s social and economic fabric, warranting focused attention and sustained action to bridge existing gaps. Addressing these issues is imperative for the current and future generations, emphasizing the need for a long-term commitment to healthcare equity in rural regions.

## Data Availability

We conducted our research using publicly available open-source data.
